# The potential regulatory role of hsa_circ_0004104 in the persistency of atrial fibrillation by promoting cardiac fibrosis via TGF-β pathway

**DOI:** 10.1186/s12872-021-01847-4

**Published:** 2021-01-09

**Authors:** Yuanfeng Gao, Ye Liu, Yuan Fu, Qianhui Wang, Zheng Liu, Roumu Hu, Xinchun Yang, Mulei Chen

**Affiliations:** Heart Center and Beijing Key Laboratory of Hypertension, Department of Cardiology, Beijing Chaoyang Hospital, Capital Medical University, 8th Gongtinanlu Rd, Chaoyang District, Beijing, 100020 China

**Keywords:** Circular RNAs, Atrial fibrillation, Progression, Biomarkers

## Abstract

**Introduction:**

The progression of paroxysmal AF (PAF) to persistent AF (PsAF) worsens the prognosis of AF, but its underlying mechanisms remain elusive. Recently, circular RNAs (circRNAs) were reported to be associated with cardiac fibrosis. In case of the vital role of cardiac fibrosis in AF persistency, we hypothesis that circRNAs may be potential regulators in the process of AF progression.

**Materials and methods:**

6 persistent and 6 paroxysmal AF patients were enrolled as derivation cohort. Plasma circRNAs expressions were determined by microarray and validated by RT-PCR. Fibrosis level, manifested by serum TGF-β, was determined by ELISA. Pathways and related non-coding RNAs involving in the progression of AF regulated were predicted by in silico analysis.

**Results:**

PsAF patients showed a distinct circRNAs expression profile with 92 circRNAs significantly dysregulated (fold change ≥ 2, p < 0.05), compared with PAF patients. The validity of the expression patterns was subsequently validated by RT-PCR in another 60 AF patients (30 PsAF and PAF, respectively). In addition, all the 5 up and down regulated circRNAs were clustered in MAPK and TGF-beta signaling pathway by KEGG pathway analysis. Among the 5 circRNAs, hsa_circ_0004104 was consistently downregulated in PsAF group (0.6 ± 0.33 vs 1.46 ± 0.41, p < 0.001) and predicted to target several AF and/or cardiac fibrosis related miRNAs reported by previous studies. In addition, TGF-β1 level was significantly higher in the PsAF group (5560.23 ± 1833.64 vs 2236.66 ± 914.89, p < 0.001), and hsa_circ_0004104 showed a significant negative correlation with TGF-β1 level (r = − 0.797, p < 0.001).

**Conclusion:**

CircRNAs dysregulation plays vital roles in AF persistency. hsa_circ_0004104 could be a potential regulator and biomarker in AF persistency by promoting cardiac fibrosis via targeting MAPK and TGF-beta pathways.

## Introduction

Atrial fibrillation (AF) is the most common type of arrhythmic disorders worldwide [1]. The estimated prevalence for AF in adults approximates to 3%, and it is even higher in the elderly and individuals with additional AF risk factors like hypertension and coronary heart disease [2]. Besides, as a progressive disease, persistent or permanent AF is reported to be associated with greater morbidity and mortality than paroxysmal AF. As we know, the current strategy to curb the progression of AF only depend on modulation of known clinical factors or complications like heart failure and hypertension [3]. Hence, better understanding of the mechanisms and pivotal mediators of this process would be of great opportunities to provide novel targets for preventing or even reversing the progression of AF.

As an emerging class of non-coding RNAs, circular RNAs (circRNAs) have been reported to be involved in various human diseases including cancers and cardiovascular disease like atherosclerosis [4], hypertension [5], and heart failure [6]. But none of the current studies have focused on their roles in the pathogenesis of atrial fibrillation. Some studies have shown a potential role of circRNAs in the process of myocardial fibrosis [7, 8], which is one of the long-recognized mechanisms of cardiac structural remodeling and thus the initiation and progression of AF [9]. Among these studies, most of the identified circRNAs are competing endogenous RNAs (ceRNAs) that act as molecular sponges for miRNAs and thus regulate gene transcription. Therefore, we hypothesize that circRNAs could be potential mediators in the process of AF progression. Based on comparing the circRNAs expression profile between paroxysmal and persistent AF, the present study aims to figure out whether circRNAs are associated with AF progression and the potential mechanism of their roles in this process.

## Materials and methods

### Study population and data collection

6 patients with paroxysmal AF (PAF) and 6 with persistent AF (PsAF) were enrolled for circRNA microarray analysis. Another 30 pairs of paroxysmal and persistent AF patients were used for RT-PCR for circRNAs and ELISA for TGF-β1. The routine clinical assessment was performed on every patient, including history, physical examination, serial 12-lead ECG monitoring, pulse oximetry, chest radiography and bedside echocardiogram. PAF was diagnosed with AF episodes that were self-terminating or converted within 7 days. Persistent PsAF was classified as AF that lasted longer than 7 days, including episodes that were terminated by cardioversion after 7 days or more. Blood samples from each consenting patient were drawn into EDTA tubes at administration and afterwards processed within 4 h before being stored at – 80 °C.

Ethics approval was obtained by the Ethics Committees of Chaoyang Hospital affiliated to Capital Medical University (2017-2-17-20). Blood sample was collected from each participant at the time of the admission. Informed consent was obtained from each subject. The methods were carried out in accordance with the relevant guidelines, including any relevant details. All analyses were carried out blinded to clinical data.

### RNA extraction, library preparation and microarray

TRIzol method (Invitrogen, Carlsbad, CA) was used to isolate total RNAs from peripheral plasma samples according to instructions. We used Nano Drop ND-1000 spectrophotometer (Nano Drop Thermo, Wilmington, DE) to evaluate the quantity and quality of RNA. Agarose gel electrophoresis were used to assess the integrity of RNA.

To remove linear RNAs and enrich circRNAs, isolated RNAs were digested with Rnase R (Epicentre, Inc.). Then, we amplified the enriched circRNAs and transcribed them into fluorescent cRNA utilizing a random priming method (Arraystar Super RNA Labeling Kit; Arraystar). RNeasy Mini Kit (Qiagen) was used to purify the labeled circRNAs. Each labeled circRNA (1 μg) was fragmented by adding 10 × Blocking Agent (5 μl) and 25 × fragmentation buffer (1 μl), after heated the mixture at 60 °C for half-hour, we added 2 × hybridization buffer (25 μl) to dilute the labeled circRNA. We dispensed hybridization solution (50 μl) into the gasket slide and assembled to the circRNA expression microarray slide. In an Agilent Hybridization Oven, the slides were incubated at 65 °C for 17 h. The hybridized arrays were washed, fixed and scanned (Agilent Scanner G2505C). The circRNA ID in circBase (http://circbase.mdc-berlin.de) were used as the formal name in the present study.

R software and Bioconductor package was used to quantile normalization and subsequent data processing. Target circRNAs were then retained for further differential analysis. Profile differences and the fold change (i.e. the ratio of the group averages) between two groups (PsAF versus PAF) for each circRNA was compared. Paired t test was used to analyze the statistical difference. CircRNAs having fold changes > 2 and p value < 0.05 were selected as the significantly differentially expressed.

### circRNA-miRNA interaction prediction

We predicted the circRNA-miRNA interaction using Arraystar’s home-made miRNA target prediction software based on TargetScan and miRanda. To establish circRNA-miRNA network, MREs on circRNAs were searched using the software, and then selected the miRNAs accordingly. Finally, top 5 miRNA candidates were determined based on miSVR scores (comprehensive modeling of miRNA targets predicts functional non-conserved and non-canonical sites).

### circRNA-miRNA-mRNA network

TargetScan, miRDB and miRTarBase were used to predict candidate target genes for miRNA. Functions and KEGG pathways of those target genes were annotated using KOBAS 3.0. Cytoscape 3.6.0 was used to illustrate circRNA-miRNA-mRNA network map.

### Quantitative real-time PCR (qPCR)

SuperScriptTM III Reverse Transcriptase (Invitrogen, Carlsbad, CA) was used for synthesizing cDNA according to manufacturer’s instructions. ViiA 7 Real-time PCR System (Applied Biosystems) was used to evaluate the expression level of the circRNAs. Specific divergent primers were designed to amplify the circular transcripts. PCR was performed in 10 μl reaction volume, including cDNA 2 μl, 2 × master mix 5 μl, forward primer (10 μM) 0.5 μl, reverse primer (10 μ M) 0.5 μl and double distilled water 2 μl. We first set pre-denaturation reaction at 95 °C for 10 min, then set the reaction at 95 °C for 10 s and at 60 °C for 60 s, repeating 40 cycles. Both target and reference (β-actin) were amplified in triplicate wells. 2^−^$\Delta \Delta$^Ct^ method was used to calculate the relative level of each circRNA.

### Enzyme linked immunosorbent assay (ELISA)

We used ELISA method with Human Transforming Growth Factor Beta 1 ELISA Kit (Elabscience Biotechnology Co.,Ltd) to measure the plasma level of TGF-β1.The ELISA kit was balanced at room temperature for 20 min according to the instruction. 100 μl plasma sample was added to reaction wells and incubated at 37 °C for 90 min. 100 µl fresh biotinylated antibody working solution was added into the sections and further incubated at 37 °C for 60 min. 100 µl fresh enzyme combination reaction working fluid was introduced to each section after washing, and incubated at 37 °C for half-hour. The plate was rinsed 3 times and added with 100 µl TetraMethyl benzidine (TMB) substrate solution, and further incubated at 37 °C in shade for 15 min. In order to terminate the reaction, the stop solution was added immediately. The optical density (OD) value of each tube was determined within 3 min using a multi-mode microplate reader (MULTISKAN EX PRIMARY EIA V. 2.3) at 450 nm, and the content of TGF-β1 in the plasma was analyzed.

### Statistical analysis

The normal distribution of continuous variables was evaluated by Kolmogorov–Smirnov test. Normally-distributed continuous variables were expressed in mean ± standard deviation and analyzed by Student's t-test. Binary or categorical variables were expressed in numbers (%). Comparison of continuous variables used independent sample t-test. Comparison of categorical variables used with Chi-square tests. Correlation between two continuous variables was calculated using linear regression. All tests were two-sided and p < 0.05 were considered to be significant. Higher threshold (fold change ≥ 2, p < 0.05) for significancy and biological replications by qPCR were carried out to control the potential false discovery of micro-array analysis. Statistical analysis was carried out using SPSS statistics v21.0, GraphPad Prism 5 (GraphPad Software, San Diego, CA).

## Results

### PsAF patients showed a distinct circRNAs expression pattern compared to PAF patients

To investigate the potential role of circRNAs in different AF types, we utilized human circRNA microarray to quantify the expression differences in peripheral blood plasma between 6 paroxysmal and 6 persistent AF patients. There were no significant differences between two groups on the clinical characteristics or AF risk factors (Table 1), which eliminate potential confounding factors affecting the circRNA expression or contributing to the AF progression.

**Table 1:** Baseline characteristics of PAF and PsAF patients underwent circRNA microarray analysis

	PsAF (n = 6)	PAF (n = 6)	p value
Age, years	59.33 ± 6.09	58.67 ± 4.89	0.838
Male, n (%)	3 (50.0)	3 (50.0)	1.000
BMI, kg/m^2^	27.25 ± 1.47	25.45 ± 2.33	0.140
Heart rate, bpm	95.50 ± 18.21	87.00 ± 18.22	0.438
CAD, n (%)	0 (0)	1 (16.7)	1.000
HTN, n (%)	5 (83.3)	4 (66.7)	1.000
DM, n (%)	2 (33.3)	1 (16.7)	1.000
Smoke, n (%)	2 (33.3)	0 (0)	0.455
AF duration, months	9.2 ± 2.7	7.8 ± 3.1	0.291
LAD, mm	38.83 ± 5.67	39.00 ± 3.90	0.954
LVEF, %	64.50 ± 4.46	68.67 ± 5.39	0.175
LVEDD, mm	46.00 ± 4.05	47.92 ± 3.01	0.374
HbA1c, %	6.18 ± 1.03	5.86 ± 0.46	0.507
LDL, mmol/L	2.80 ± 0.42	2.87 ± 1.58	0.922
Creatine, µmol/L	64.98 ± 20.70	63.02 ± 17.55	0.863
BNP, pg/ml	1336.17 ± 806.4	1211.02 ± 741.18	0.385
β-blockers, n (%)	4 (66.7)	3 (50.0)	1.000

Data are number (%) and mean ± SD

PsAF, persistent atrial fibrillation; PAF, paroxysmal atrial fibrillation; BMI, body mass index; bpm, beats per minute; CAD, coronary artery disease; HTN, hypertension; DM, diabetes mellitus; AF, atrial fibrillation; LAD, left atrial diameter; LVEF, left ventricular ejection fraction; LVEDD, left ventricular end diastolic diameter; HbA1c, glycosylated hemoglobin; LDL, low density lipoprotein

**Fig. 1:**
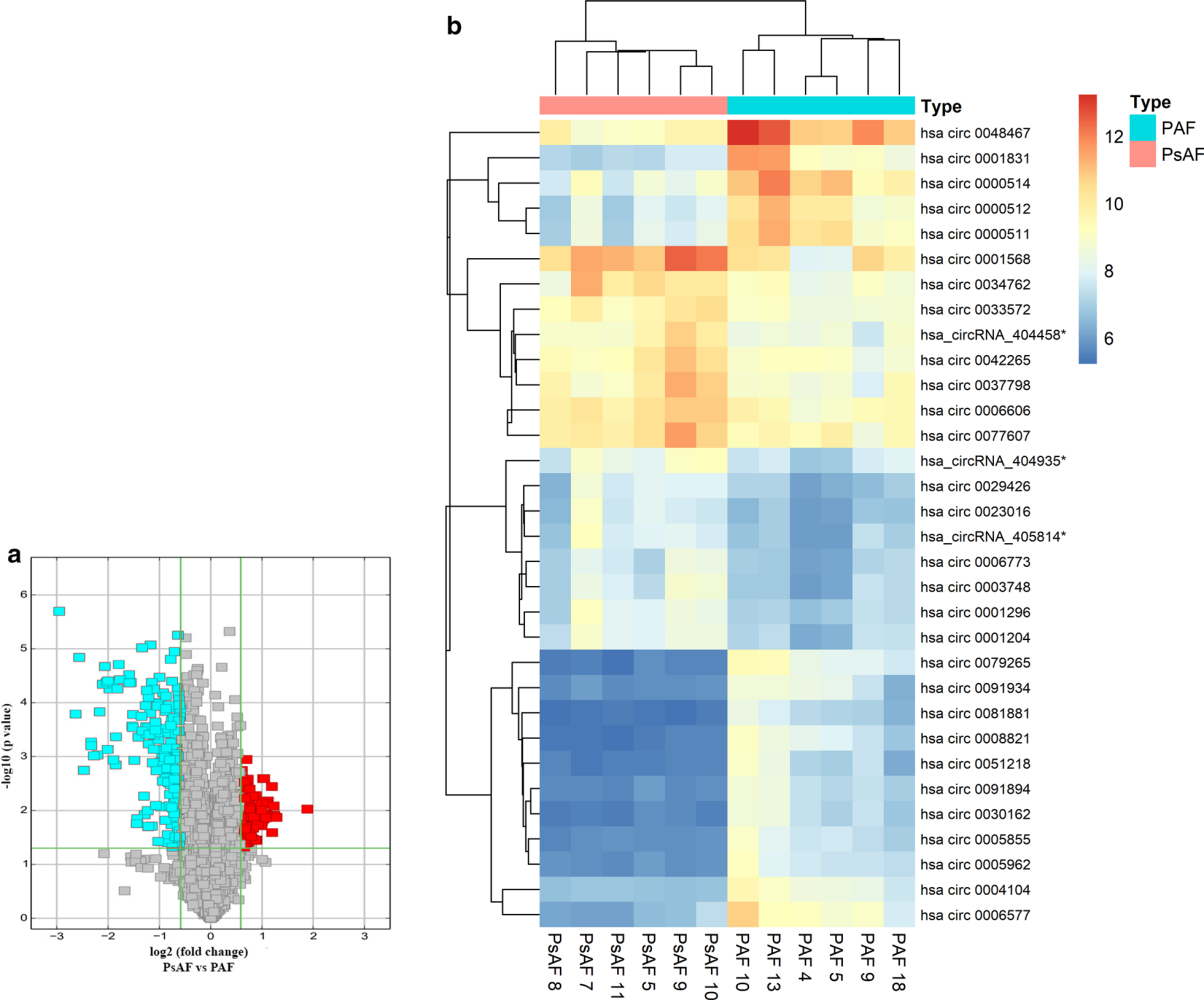
Microarray results showed differential expression of circular RNA in two AF groups. **a** Volcano plots are used for visualizing differential expression between the two different conditions. The vertical lines correspond to 2.0 fold (log2 scaled) up and down, respectively, and the horizontal line represents the p value of 0.05 (− log scaled). The red points in the plot represent up regulated circRNAs with statistical significance, while the cyan points represent down regulated circRNAs with statistical significance. **b** The hierarchically clustered heatmap of partial differentially expressed circRNAs. ‘Orange’ indicates high relative expression, and ‘blue’ indicates low relative expression

As seen in the volcano plots (Fig. 1a) and heatmap (Fig. 1b), the circRNA expression profile was distinct between PAF and PsAF patients. To be specific, 92 circRNAs were significantly dysregulated (fold change ≥ 2, p < 0.05), with 76 down-regulated circRNAs and 16 circRNAs up-regulated in PsAF group compared to PAF group.

### Biological replication confirmed the consistency of the microarray results

**Fig. 2:**
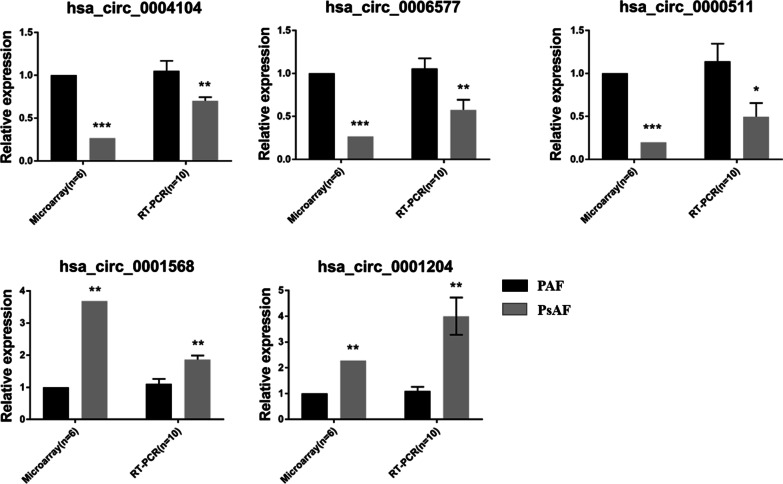
Results of qPCR validation for dysregulated circular RNAs. hsa_circ_0004104, hsa_circ_0006577, hsa_circ_0000511, hsa_circ_0001568, hsa_circ_0001204, remained statistically significant difference in expression levels. *p < 0.05; **p < 0.01; ***p < 0.001

We selected > twofold change and p < 0.01 in circRNAs for more stringent significance. Besides, potential candidates were excluded when effective primers could not be designed. The expression of 7 circRNAs were assessed by qPCR in another 20 subjects (10 PAF and 10 PsAF, respectively) recruited for validation (biological replication). For these circRNAs, qPCR replicated five out of the seven circRNAs (*hsa_circ_0006577*, *hsa_circ_0004104*, *hsa_circ_0001568*, *hsa_circ_0001204*, *hsa_circ_0000511*) as statistically significant in the samples, while the other two showed a too low abundance to quantify (Fig. 2).

### Multiple miRNAs targeting TGF-beta pathways were predicted to be sponged by the dysregulated circRNAs

**Fig. 3:**
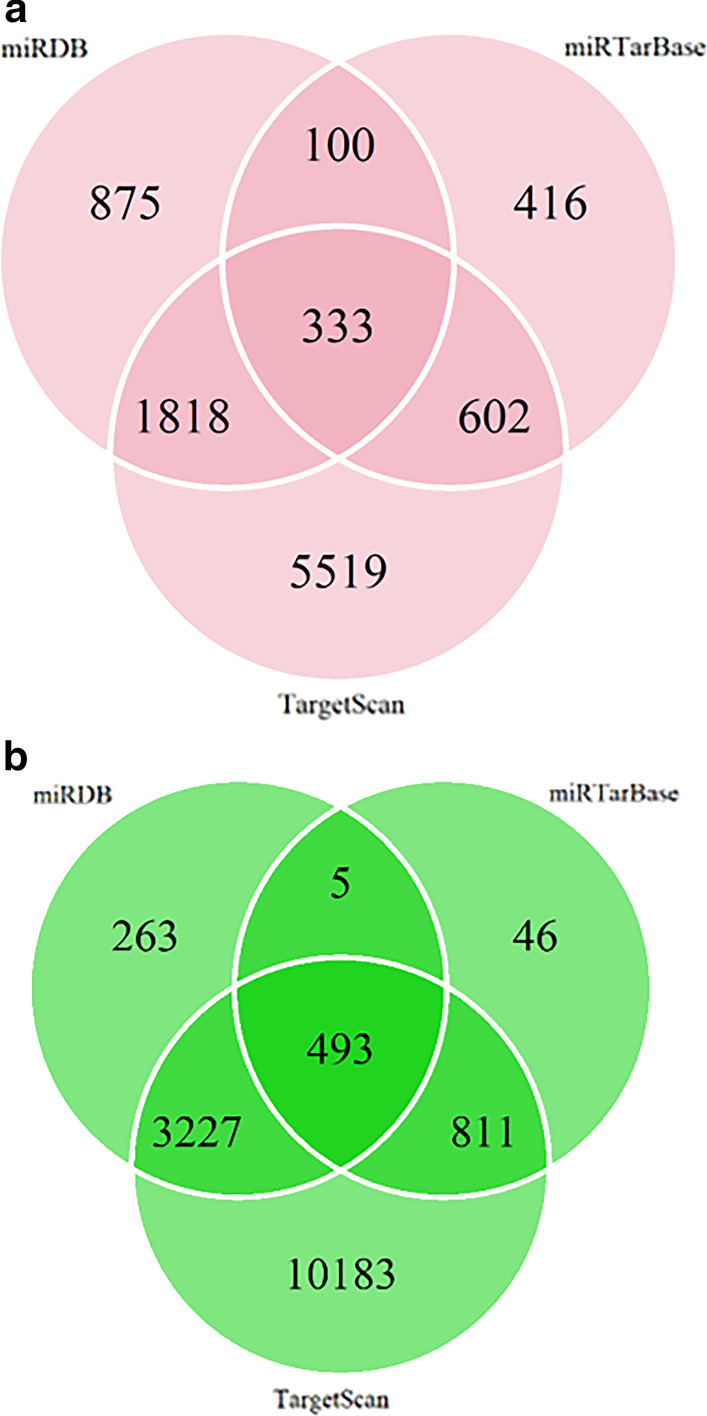
Venn diagram of miRNA target genes predicted. **a** Venn diagram of miRNA target genes predicted from up regulated circRNAs using TargetScan, miRanda, and miRTarBase. **b** Venn diagram of miRNA target genes predicted from down regulated circRNAs using TargetScan, miRanda, and miRTarBase

**Fig. 4:**
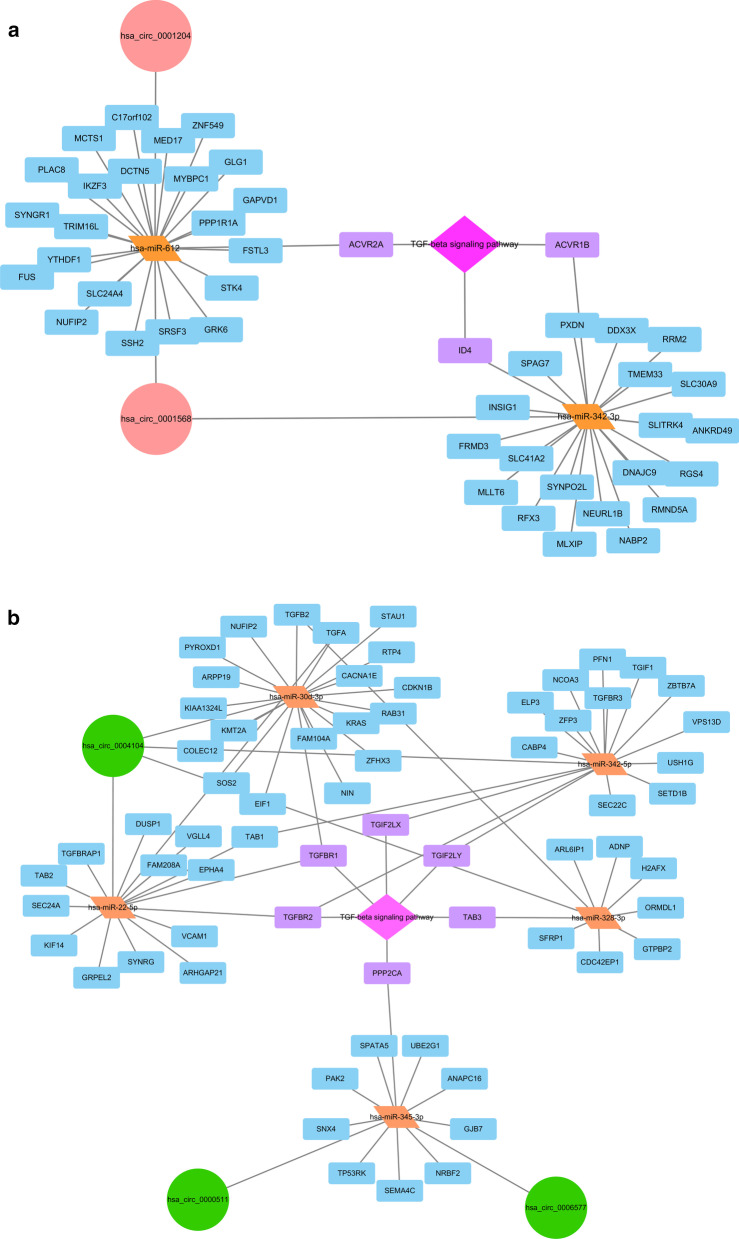
Prediction of target genes and circRNA-miRNA-mRNA network. **a** circRNA-miRNA-mRNA network for up regulated circRNAs. Genes targeting TGF-β1 signaling pathway were illustrated with purple color. **b** circRNA-miRNA-mRNA network for down regulated circRNAs. Genes targeting TGF-β1 signaling pathway were illustrated with purple color

To elucidate the function of selected circRNAs, targeted miRNAs were predicted with Arraystar's home-made miRNA target prediction software based on TargetScan and miRanda (see details in method part). Targeted miRNAs were ranked according to the mirSVR scores, thereby identifying the top 5 ranking candidates for further analysis. Top 5 predicted miRNAs of selected circRNAs were used for prediction of target genes using TargetScan, miRanda, and miRTarBase combined. For up-regulated circRNAs, hsa_circ_0001568 and hsa_circ_0001204, 333 target genes were predicted in all three databases (Fig. 3a). For down-regulated circRNAs, hsa_circ_0006577, hsa_circ_0004104, and hsa_circ_0000511, 493 target genes were incorporated in all three databases (Fig. 3b). CircRNA-miRNA-mRNA network and genes directly targeting TGF-β1 signaling pathway were illustrated on Fig. 4a, b. 3 genes (ACVR2A, ACVR1B, and ID4) from down-regulated circRNAs and six genes (TGIF2LX, TGIF2LY, TAB3, PPP2CA, TGFBR1, and TGFBR2) from up-regulated circRNAs targeted TGF-beta signaling pathway.

**Fig. 5:**
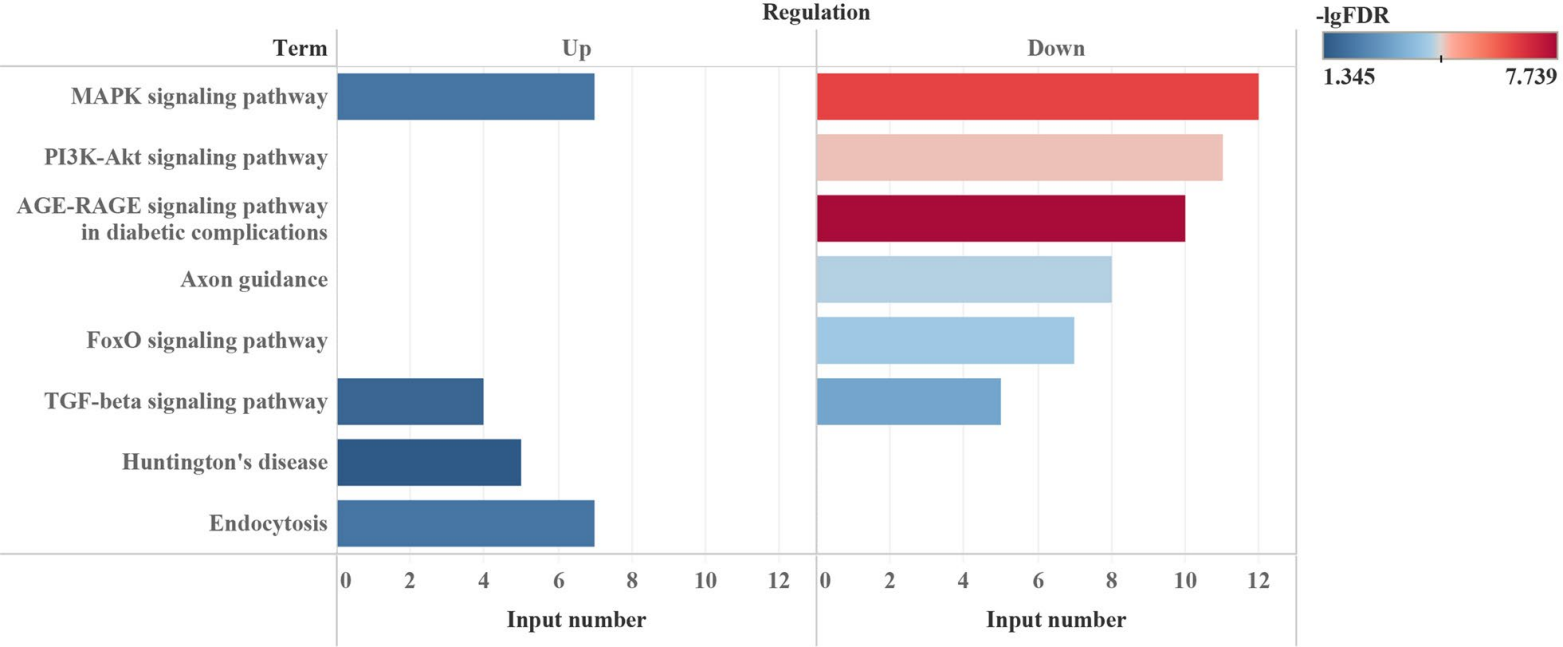
Signaling pathway for dysregulated circRNAs predicted from miRNA target genes. Both up and down regulated circRNAs clustered in MAPK and TGF-beta signaling pathway. Input number refers to number of gene count clustered in each term. Color shows-lgFDR as an attribute

Functional annotations were carried-out with KOBAS 3.0 to generated significant GO term and KEGG signaling pathways (FDR < 0.05). For up regulated circRNAs, GO annotations were mainly clustered in nucleus, cytoplasm, and nucleoplasm in cellular component (CC); peptidyl-tyrosine dephosphorylation, positive regulation of transcription from RNA polymerase II promoter, and positive regulation of transcription, DNA-templated in biological process (BP). And protein tyrosine phosphatase activity, protein binding, and protein tyrosine/serine/threonine phosphatase activity in molecular function (MF). KEGG pathways for those miRNAs clustered in MAPK signaling pathway, Endocytosis, TGF-beta signaling pathways and Huntington's disease. For down regulated circRNAs, GO annotations were mainly clustered in focal adhesion, membrane, and extracellular matrix in CC; pathway-restricted SMAD protein phosphorylation, transforming growth factor beta receptor signaling pathway, and collagen fibril organization in BP. In addition, SMAD binding, type II transforming growth factor beta-receptor binding, and transforming growth factor beta-activated receptor activity in MF. KEGG pathways for those miRNAs clustered in AGE-RAGE signaling pathway in diabetic complications, MAPK signaling pathway, ErbB signaling pathway, PI3K-Akt signaling pathway, Axon guidance, FoxO signaling pathway, and TGF-beta signaling pathway. As was shown in Fig. 5, both up and down regulated circRNAs clustered in MAPK and TGF-beta signaling pathway.

### TGF-β1 pathway was significantly activated in PsAF patients

**Fig. 6:**
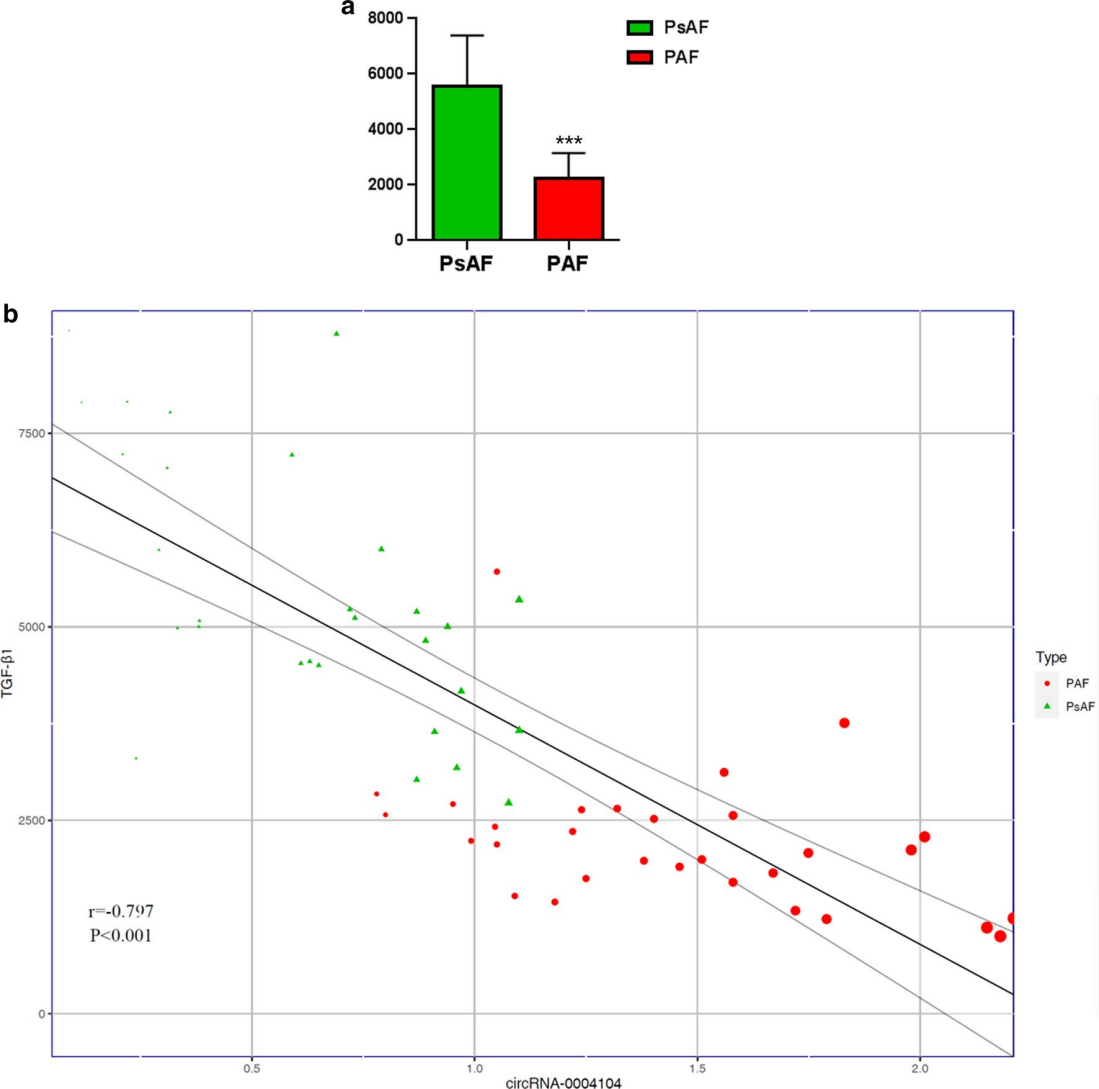
Expression levels of TGF-β1 in two AF groups. Correlation between hsa_circ_0004104 and TGF-β1. **a** PsAF patients had significantly higher level of TGF-β1 than PAF, p < 0.001. **b** Negative correlation between hsa_circ_0004104 and TGF-β1 verified by linear regression (r = −0.797, p < 0.001). Larger bubble size referred to higher level of plasma TGF-β1. ***p < 0.001

The genomic locus of exonic hsa_circ_0004104 was on chromosome 5. The top 5 predicted miRNA targets of hsa_circ_0004104 were hsa-miR-194-3p, hsa-miR-22-5p, hsa-miR-342-5p, hsa-miR-328-3p, and hsa-miR-30d-3p. Among them, hsa-miR-328-3p and hsa-miR-30d-3p were reported to correlate with TGF-beta signaling pathway and structural remodeling in atrial fibrillation. Besides, serum TGF-β1 level has been recognized as surrogate of cardiac fibrosis. In view of that the results above, we further quantified the serum hsa_circ_0004104 and TGF-β1 level in an expanded sample size of 30 persistent vs 30 paroxysmal AF patients. Two groups showed no significant difference in clinical profiles (Table2), while patients with PsAF expressed significantly lower level of hsa_circ_0004104 (0.6 ± 0.33 vs 1.46 ± 0.41, p < 0.001). As seen in Fig. 6a, TGF-β1 level was significantly higher in the PsAF group (5560.23 ± 1833.64 vs 2236.66 ± 914.89, p < 0.001), indicating a potential higher level of cardiac fibrosis. In addition, hsa_circ_0004104 showed a significant reverse correlation with TGF-β1 level (r = − 0.797, p < 0.001) by Linear regression analysis (Fig. 6b), in both of the PAF (r = − 0.421, p = 0.02) and PsAF (r = − 0.665, p < 0.001) patients.

**Table 2:** Demographics of PAF and PsAF patients for circRNA expression validation and serum TGF-β1 level quantification

	PsAF (n = 30)	PAF (n = 30)	p value
Age, years	64 ± 9.46	63.67 ± 5.74	0.87
Male, n (%)	22 (73.3)	21(70)	0.774
BMI, kg/m^2^	27.28 ± 3.92	26.49 ± 4.07	0.452
Heart rate, bpm	88.77 ± 17.51	83.37 ± 23.88	0.322
CAD, n (%)	9 (30)	5 (16.7)	0.222
HTN, n (%)	18 (60)	20 (66.7%)	0.592
DM, n (%)	7 (23.3)	9 (30)	0.559
Smoke, n (%)	8 (26.7)	9 (30)	0.627
AF duration, months	14.3 ± 2.6	8.4 ± 2.9	0.056
LAD, mm	42.43 ± 4.35	40.72 ± 4.65	0.61
LVEF, %	63 ± 7.77	63.53 ± 7.46	0.787
LVEDD, mm	48.8 ± 4.05	49.2 ± 4.31	0.712
HbA1c, %	6.43 ± 1.02	5.81 ± 1.9	0.126
LDL, mmol/L	2.25 ± 0.84	2.56 ± 0.82	0.156
Creatine, µmol/L	74.15 ± 16.19	71.56 ± 17.13	0.55
BNP, pg/ml	1506.82 ± 719.46	1142.11 ± 782.98	0.185
β-blockers, n (%)	21 (70)	20 (66.7)	0.603
ACEI/ARB, n (%)	11 (36.7)	13 (43.3)	0.511
hsa_circ_0004104	0.6 ± 0.33	1.46 ± 0.41	< 0.001
TGF-β1, pg/ml	5560.23 ± 1833.64	2236.66 ± 914.89	< 0.001

Data are number (%) and mean ± SD

PsAF, persistent atrial fibrillation; PAF, paroxysmal atrial fibrillation; BMI, body mass index; bpm, beats per minute; CAD, coronary artery disease; HTN, hypertension; DM, diabetes mellitus; AF, atrial fibrillation; LAD, left atrial diameter; LVEF, left ventricular ejection fraction; LVEDD, left ventricular end diastolic diameter; HbA1c, glycosylated hemoglobin; LDL, low density lipoprotein. BNP, brain natriuretic peptide; ACEI, angiotensin-converting enzyme inhibitor; ARB, agiotensin Receptor Blocker; TGF-β1, Transforming growth factor-beta 1

## Discussion

Atrial fibrosis has long been known to associate with the persistence of AF in both animal models and human studies [10]. The present study provided further evidence for this hypothesis from the perspective of non-coding RNAs, to be specific, circRNAs by a hypothesis free measure (microarray). There are several strengths of the present study: (1) this was the first interrogation of the human plasma circRNAs signature and its potential functional impact on the progression of AF; (2) MAPK and TGF-beta signaling pathway were indicated to involve in the progression of atrial fibrillation; (3) circRNAs could be involved in the AF progression by promoting cardiac fibrosis.

The natural history of AF was progression from self-terminating paroxysms to a more sustained state [10]. Several clinical variables have been previously associated with AF progression, including age, diabetes, valvular heart disease, heart failure, heart rate and an increased left atrial (LA) enlargement [11]. Atrial Structural remodeling or fibrotic atrial cardiomyopathy was reported to be related to paroxysmal AF progression [12]. The presence of interstitial fibrosis could lead to changes in cellular coupling resulting in spatial ‘non-uniform anisotropic’ impulse propagation atrial activation abnormalities. This process underlies the initiation and perpetuation of AF [13]. Fibrotic structural remodeling could be attributed to activation of several pathways fine-tuned by different types of modulating factors. Mitogen-activated protein kinase (MAPK) signaling pathway was crucial in the process of atrial fibrosis. Tsai CF et al. reported that aldosterone significantly increased activation of p38 MAPK pathways and increased expression of fibrotic marker proteins like transforming growth factor (TGF)-β1 and α-smooth muscle actin (SMA) [14]. In addition, Jinqi Fan have reported that over expression of ACE2 would inhibit MAPK activation, decrease TGF-β1and attenuate atrial fibrosis [15]. Experimental studies have also demonstrated the possible roles of miRNAs in AF progression [16]. TakeshiSoeki et al. studied intra cardiac blood samples from AF and sinus rhythm and discovered higher level of miRNA-328 in AF patients. Level of miR-328 showed a positive correlation with the LA voltage zone index and might be involved in the process of atrial remodeling [17]. Another micro RNA, miR-30 was found to elevate in cardiomyocytes from AF patients [18], and was related to diffuse myocardial fibrosis [19]. MiR-328 was reported to target CACNA1C/Cav1.2 [20] and miR-30d could target KCNJ3/Kir3.1 [18], which could lead to adverse electrical remodeling in atrial fibrillation. In the present study, the circRNAs signature showed a significant different pattern between PAF and PsAF, which indicate that circRNAs expression change could involve in the process of AF progression from paroxysmal to persistent AF. In addition, the significantly down-regulated circRNA hsa_circ_0004104 was predicted to target these two AF-relating miRNAs (miR-30 and miR-328), indicating that the potential functional impact of circRNAs could be its upstream regulatory effect.

A major mechanism of circRNAs in the regulation of cell function was to act as miRNA sponges [21]. Recently reported circRNA_010567 could promote myocardial fibrosis via suppressing miR-141 by targeting TGF-β1 [8]. Similarly, over-expression of circRNA_000203 was reported to have the capacity of eliminating the anti-fibrosis effect of miR-26b-5p in cardiac fibroblasts [7]. The results of our study added yet another example of the sponge effect of circRNAs in the process of disease. Besides, due to its stability endowed by its molecular structure, circRNAs had been proposed to be potential biomarkers for cardiovascular diseases [22]. In the present study, we found that the circRNA hsa_circ_0004104 was down-regulated both by microarray and RT-PCR validation. In addition, it showed a reverse association with the known marker of atrial fibrosis TGF- β1. These in all indicated a potential biomarker for the circRNA hsa_circ_0004104 in the prediction of AF progress.

The top 5 miRNAs targeted by hsa_circ_0004104 were all reported to affect the TGF-β1 pathway. MiR-328 from cardiomyocytes could enhance collagen deposition and provoke cardiac fibrosis by activation of TGF-β1 pathway in a mouse AF model [23]. Meantime, demethylation of miR-30d precursor gene and restoration of miR-30d would inhibit effects of TGF-β1. Other miRNAs that were significantly targeted by the circRNA hsa_circ_0004104, like miR-29 [24], miR-19a-3p/19b-3p [25], and miR-208a [26] were all reported to modulate TGF-β1. Fortunately, TGF-β1 was a secretory protein which could be detected in serum. We further investigated the serum level of TGF-β1 to lend some evidence for the functional impact of the circRNA hsa_circ_0004104 in the progression of AF. It turned out that hsa_circ_0004104 correlated with the expression of serum TGF-β1, which added a concrete strength to the role of hsa_circ_0004104 in the TGF-beta signaling pathway predicted by in silico analysis and thus AF progression.

Recently, a study by Wang LY profiled transcriptome-wide circRNAs expression in peripheral blood mononuclear cells (PBMCs) of patients with and without coronary artery disease (CAD), after validating in a large cohort, two circRNAs, hsa_circ_0004104 and hsa_circ_0001879, were found to be diagnostic biomarkers for CAD [27]. The study also demonstrated that hsa_circ_0004104 may contribute to the pathogenesis of CAD by upregulating of atherosclerosis-susceptible genes, such as IDO1, MMP8, CD40, and downregulating of anti-atherosclerosis genes, such as ApoA I, RNASE1 [27]. However, the mechanisms by which hsa_circ_0004104 regulate the progression of PAF to PsAF remain elusive. We found the genomic locus of exonic hsa_circ_0004104 was on chromosome 5, nearby the secreted protein acidic and rich in cysteine (SPARC) gene, and SPARC was proved to be an important role in cardiac repair and fibrosis previously [28]. Hsa_circ_0004104 may regulate the progression of AF by affecting the expression of SPARC gene, this hypothesis, however, needs further experimental validation.

The present study suffers from several limitations: (1) there were no in vitro study of the candidate circRNA regarding its effects in the atrial cells; (2) the sample size of the validation groups was relatively small; (3) to establish the biomarker role of hsa_circ_0004104 in predicting AF progression, a cohort study following the PAF patients was needed. Despite of these drawbacks, the present study still provides novel proposals and preliminary data that shed light on the role of circRNAs in the important but under-researched issue of AF persistency.

## Conclusion

To sum up, the present study found significant differentiated expression patterns of circRNAs between paroxysmal and persistent AF. This indicates the potential role of circRNAs dysregulation in the process of cardiac fibrosis as well as AF progression. Besides, the results of in silico* analysis and *in vivo studies also indicated that circRNA hsa_circ_0004104 could be a potential regulator and biomarker in the persistency of AF by promoting cardiac fibrosis via targeting MAPK and TGF-beta pathway.

## Data Availability

The datasets generated and/or analysed during the current study are not publicly available due to the restrictions by the Beijing Chaoyang Hospital but are available from the corresponding author on reasonable request.

## Abbreviations

AF: Atrial fibrillation
PAF: Paroxysmal AF
PsAF: Persistent AF
MiRNA: MicroRNA
CircRNA: Circular RNA
CeRNA: Competing endogenous RNA
qPCR: Quantitative real-time PCR
ELISA: Enzyme linked immunosorbent assay
TMB: TetraMethyl benzidine
OD: Optical density
LA: Left atrial
MAPK: Mitogen-activated protein kinase
TGF-β: Transforming growth factor-beta
SMA: α-Smooth muscle actin
PBMCs: Peripheral blood mononuclear cells
CAD: Coronary artery disease
SPARC: Secreted protein acidic and rich in cysteine
